# The passive leg raising test to guide fluid removal in critically ill patients

**DOI:** 10.1186/s13613-016-0149-1

**Published:** 2016-05-20

**Authors:** Xavier Monnet, Flora Cipriani, Laurent Camous, Pierre Sentenac, Martin Dres, Evguenia Krastinova, Nadia Anguel, Christian Richard, Jean-Louis Teboul

**Affiliations:** Service de réanimation médicale, Hôpital de Bicêtre, Hôpitaux universitaires Paris-Sud, 78, rue du Général Leclerc, 94270 Le Kremlin-Bicêtre, France; Faculté de médecine Paris-Sud, Inserm UMR S_999, Univ Paris-Sud, 63, rue Gabriel Péri, 94270 Le Kremlin-Bicêtre, France; Service de santé publique, Hôpital de Bicêtre, Hôpitaux universitaires Paris-Sud, 78, rue du Général Leclerc, 94270 Le Kremlin-Bicêtre, France; Service de réanimation médicale, Centre Hospitalier Universitaire de Bicêtre, 78, rue du Général Leclerc, 94270 Le Kremlin-Bicêtre, France

**Keywords:** Passive leg raising, Renal replacement therapy, Ultrafiltration, Volume responsiveness, Acute kidney injury, Hypotension

## Abstract

**Background:**

To investigate whether haemodynamic intolerance to fluid removal during intermittent renal replacement therapy (RRT) in critically ill patients can be predicted by a passive leg raising (PLR) test performed before RRT.

**Methods:**

We included 39 patients where intermittent RRT with weight loss was decided. Intradialytic hypotension was defined as hypotension requiring a therapeutic intervention, as decided by the physicians in charge. Before RRT, the maximal increase in cardiac index (CI, pulse contour analysis) induced by a PLR test was recorded. RRT was then started.

**Results:**

Ultrafiltration rate was similar in patients with and without intradialytic hypotension. Thirteen patients presented intradialytic hypotension, while 26 did not. In patients with intradialytic hypotension, it occurred 120 min [interquartile range 60–180 min] after onset of RRT. In the 26 patients without intradialytic hypotension, the PLR test induced no significant change in CI. Conversely, in patients with intradialytic hypotension, PLR significantly increased CI by 15 % [interquartile range 11–36 %]. The PLR-induced increase in CI predicted intradialytic hypotension with an area under the ROC curve of 0.89 (95 % interval confidence 0.75–0.97) (*p* < 0.05 from 0.50). The best diagnostic threshold was 9 %. The sensitivity was 77 % (95 % confidence interval 46–95 %), the specificity was 96 % (80–100 %), the positive predictive value was 91 % (57–100 %), and the negative predictive value was 89 % (72–98 %). Compared to patients without intolerance to RRT, CI decreased significantly faster in patients with intradialytic hypotension, with a slope difference of −0.17 L/min/m^2^/h.

**Conclusion:**

The presence of preload dependence, as assessed by a positive PLR test before starting RRT with fluid removal, predicts that RRT will induce haemodynamic intolerance.

**Electronic supplementary material:**

The online version of this article (doi:10.1186/s13613-016-0149-1) contains supplementary material, which is available to authorized users.

## Background

Removing fluid is a common therapeutic option in patients with acute circulatory failure and/or acute respiratory distress syndrome (ARDS), especially at the late phase of disease [1, 2]. This is based on the evidence that the cumulative fluid balance is an independent factor that increases mortality in patients with septic shock [3] and ARDS [4], especially in the case of acute kidney injury [5]. Fluid overload may impair the function of many organs, including the kidneys [6]. Along with the prediction of preload unresponsiveness, which prevents administration of ineffective fluid boluses, fluid removal contributes to reducing the fluid balance. Compared to a liberal strategy of fluid administration, a conservative strategy including fluid removal prompted extubation in ARDS patients [7]. The net fluid removal by renal replacement therapy (RRT) was shown to decrease the intra-abdominal pressure, the cardiac preload and the extravascular lung water [8].

However, the risk of fluid removal is to remove too much fluid and to impair the haemodynamic condition. Intradialytic hypotension is obviously deleterious [9] and alters renal recovery in patients with acute renal failure [10, 11]. Also, in cases when it leads to premature termination of RRT, intradialytic hypotension reduces the dose of dialysis and the volume of fluid removal [9, 12].

Decrease in cardiac output as an adverse effect of excessive fluid removal should occur in the case of preload dependence, i.e. when changes in cardiac preload physiologically result in changes in cardiac output. The aim of this study was to test whether the presence of preload dependence can predict intolerance to fluid removal performed with ultrafiltration in patients under RRT. Passive leg raising (PLR) was used to test preload dependence.

## Patients and methods

### Patients

The study was conducted in the 15-bed medical intensive care of a university hospital. It was approved by the institutional review board of our institution (Comité pour la Protection des Personnes Ile-de-France VII). Informed consent was obtained from all patients or next of kin.

Patients were enrolled under the following conditions:

1. Episode of acute circulatory failure, in its acute or resolution phase, defined by (1) systolic blood pressure <90 mmHg (or a decrease >50 mmHg in previously hypertensive patients) or the need for norepinephrine, (2) urine output <0.5 mL/kg/h for at least 2 h, (3) tachycardia >100 beats/min, (4) skin mottling or (5) blood lactate >2 mmol/L. The resolution phase of circulatory failure was defined by a decrease of the dose of norepinephrine for more than 24 h.
2. Monitoring by a PiCCO2 device (Pulsion Medical Systems, Munich, Germany) already in place.
3. Decision by the physician in charge to perform intermittent RRT with weight loss.
4. Haemodynamic stability, as defined by no volume expansion, no increase in the dose of catecholamines and no change in mean arterial pressure >10 mmHg during the last 3 h.

Patients were excluded if they presented intracranial hypertension (which is a contraindication to the PLR test), if RRT was interrupted for non-haemodynamic reasons and if ventilation and sedation modalities were changed during the study period.

### Transpulmonary thermodilution and pulse contour analysis

All patients had an internal jugular vein catheter and a thermistor-tipped arterial catheter (PV2024 Pulsion Medical Systems, Munich, Germany) in the femoral artery connected to the PiCCO2 device. The dialysis catheter was in the femoral position, contralateral to the arterial catheter. Three cold boluses were used when performing transpulmonary thermodilution [13]. This allowed us to measure cardiac index (CI) (through transpulmonary thermodilution and pulse contour analysis), global end-diastolic volume and extravascular lung water (both through transpulmonary thermodilution) [14].

Global end-diastolic volume corresponds to the volume of the four cardiac chambers at end diastole and is a marker of cardiac preload [15]. Extravascular lung water is the volume of water that is contained in the interstitium, the intracellular compartment, the lymphatic fluid, the surfactant and the alveoli [16, 17]. Echocardiography was performed at the time of inclusion.

It has been already reported that extracorporeal circulation of blood during RRT does not alter the reliability of transpulmonary thermodilution measurements [18, 19], even for blood pump flows as high as 350 mL/min [19], even though this has been reported during continuous and not intermittent RRT. All values were indexed by the ideal and not the actual body weight, so that indexation in itself could not change the variables due to the simple weight loss induced by fluid removal.

### RRT modalities

Intermittent RRT was performed according to the current practice of our unit. It was performed with the AK200-TS device (Gambro, Lund, Sweden) by using AN69-ST membranes (Hospal, Meyzieu, France). In our unit, besides intermittent RRT, we also use continuous RRT, which is preferred in cases of haemodynamic instability. Patients receiving continuous RRT were not included in the study.

Patients had a 14F dual-lumen catheter inserted in the femoral or jugular veins (with 25- and 14-cm catheter lengths, CS-26142-F and CS-12142-F, Arrow Intl, Reading, PA, respectively). Both lines of the circuit filled with heparinised 0.9 % saline were simultaneously connected to the catheter. Circuit anticoagulation was performed with enoxaparin (4000 IU in the circuit at the beginning of dialysis), except if unfractionated heparin was already intravenously administered for another reason. In the case of high haemorrhagic risk, anticoagulation was not performed, but the circuit was flushed with 100 mL of saline every 30 min. No fluid was systematically infused at the induction of haemodialysis.

Purified (reverse osmosis) water and bicarbonate-based dialysate were used. According to our practice, dialysate sodium concentration was set at 145 mmol/L, except in the case of severe dysnatremia. Potassium concentration was set at 3 mmol/L, except in the case of severe hyperkalaemia. Dialysate temperature was 36 °C. Dialysate flow rate was 500 mL/min in all sessions, and blood flow rate was 200–300 mL/min. The duration of intermittent RRT and the volume of fluid removed were planned by the physicians in charge of the patient.

### Study design

Patients were included when RRT with ultrafiltration was planned and the RRT modalities were fixed. The study protocol stipulated that these modalities must not be changed by the participation of the patient to the study and by the results of the PLR test.

At baseline, before connecting the patient to the RRT circuit, a first set of haemodynamic variables was recorded, including heart rate, blood pressure, CI (measured by transpulmonary thermodilution), global end-diastolic volume and extravascular lung water. A PLR test was then performed as it has been previously described [20]. Briefly, the patient was moved from the semi-recumbent position to a PLR position, with legs elevated at 45° and the trunk horizontal. This was achieved by moving the bed without touching the patient. The peak CI value (measured by pulse contour analysis) during the PLR test was recorded. It is usually attained within one minute [21]. Transpulmonary thermodilution was not performed during PLR because, due to the time required by the repeated bolus injections, it could miss the maximal change in CI, which may decrease after having reached its peak value in some patients [20].

The patient was moved back to the semi-recumbent position. After stabilisation of CI, intermittent RRT was started. Immediately after the beginning of RRT (H0) and at each of the *n* hours until the end of RRT (H1 to Hn), haemodynamic variables were recorded again, including heart rate, blood pressure, CI (measured by transpulmonary thermodilution), global end-diastolic volume and extravascular lung water. Clinicians in charge were not blind for the result of the PLR test.

### Intolerance to intermittent RRT

Haemodynamic intradialytic hypotension was defined as the occurrence of one episode of hypotension requiring one or more of the following interventions from the clinicians in charge: interruption of fluid removal, introduction of norepinephrine or increase in its dose, administration of volume expansion or interruption of RRT. Hypotension was defined as a mean arterial pressure lower than 65 mmHg [22], except in patients with a previous medical history of chronic hypertension. In this case, hypotension was defined by a mean arterial pressure lower than 80 mmHg. In all patients, a set of haemodynamic measurements was recorded at the time of hypotension before any further intervention. In patients where RRT was interrupted, a set of haemodynamic measurements was also recorded immediately after blood restitution. For better clarity of data presentation in patients with intradialytic hypotension, analysis was stopped at the time of hypotension, even if this did not lead to the interruption of RRT (i.e. if hypotension led to the introduction/increase in the dose of norepinephrine or the stop of fluid removal).

### Statistical analysis

The normality of data distribution was tested with the Anderson–Darling test. Data are expressed as median [interquartile range] or *n* (frequency in %), as appropriate. The primary analysis consisted in predicting the occurrence of intradialytic hypotension by the means of the PLR test performed before RRT. Receiver operating characteristic (ROC) curves were constructed to test the ability of PLR-induced changes in CI and of CI at baseline to predict intradialytic hypotension. The areas under the ROC curves (AUC) were compared using the DeLong’s test. AUC, sensitivities, specificities, positive and negative predictive values are expressed as the values [95 % confidence interval]. The best value of PLR-induced changes in CI and arterial pulse pressure (PP) for predicting intradialytic hypotension was determined as the one providing the best Youden index.

A secondary analysis consisted in describing the time course of different variables in patients with and without intolerance to RRT. The dynamics of the variables was modelled using linear mixed-effect models with a random intercept and slope and compared [23]. Mixed-effect models that we used are currently the best way to explore longitudinal data with repeated measurements over time. Mixed models take into account the correlation between measurements in a given subject and more importantly use the whole information (i.e. all the measurements), providing a greater power than when the outcome is dichotomised as, for example, in logistic regression analysis. The models included both fixed and random effects for the intercept and slope. In multivariate analysis, the model was adjusted on age, fluid removal and initial systolic arterial pressure.

The sample size was estimated by considering a predicted mean value of CI at a baseline of 3 L/min/m^2^, a standard deviation of CI at a baseline of 1 L/min/m^2^, a PLR-induced change in CI of 20 % in patients with intradialytic hypotension [24] and an incidence of intradialytic hypotension of 33 %, with an α-risk of 5 % and a β-risk of 20 %. Eventually, the sample size was estimated to be 26 cases of well-tolerated RRT and 13 cases poorly tolerated RRT. Statistical analysis was performed with MedCalc 8.1.0.0 software (Mariakerke, Belgium). Mixed model analyses were performed with STATA software (release 13; StataCorp., College Station, Texas, USA).

## Results

### Patient characteristics

Four patients were excluded because RRT was interrupted due to filter clotting and three others because the dose of sedative drugs was increased during the study period.

Among the 39 remaining patients, six were under chronic intermittent haemodialysis before ICU admission. Eight patients had a renal transplant. No patient received antihypertensive treatment at the time of the study. The characteristics of the 26 patients without intradialytic hypotension and of the 13 patients with intradialytic hypotension are described in Table 1. RRT settings in both groups are shown in Table 2. No patient presented clinical signs of intra-abdominal hypertension. No patient received dobutamine. Mortality in the intensive care unit was 54 % in both groups of patients.

**Table 1:** Patient characteristics

	Patients without intradialytic hypotension (*n* = 26)	Patients with intradialytic hypotension (*n* = 13)	*p* value
Age (years)	63 [49–69]	63 [54–80]	0.17
Male gender (no, %)	17 (65 %)	8 (59 %)	0.99
Simplified Acute Physiology Score II	41 [46–55]	49 [45–53]	0.49
Previous chronic renal failure (no, %)	10 (40 %)	5 (38 %)	0.82
Previous chronic arterial hypertension (no, %)	8 (30 %)	5 (40 %)	0.79
Aetiology of acute circulatory failure (no, %)			
Septic	20 (77 %)	10 (77 %)	0.69
Cardiogenic	6 (23 %)	3 (23 %)	0.69
Incidence of shock diagnosis criteria at the initial phase			
Systolic arterial pressure <90 mmHg	26 (100 %)	13 (100 %)	–
Decrease >50 mmHg in previously hypertensive patients	0 (0 %)	1 (8 %)	0.68
Need for norepinephrine administration	26 (100 %)	13 (100 %)	–
Urine output <0.5 mL/kg/h for at least 2 h	26 (100 %)	12 (92 %)	0.68
Heart rate >100 beats/min	20 (77 %)	13 (100 %)	0.16
Skin mottling	8 (31 %)	6 (46 %)	0.57
Blood lactate >2 mmol/L	26 (100 %)	11 (85 %)	0.21
Left ventricular ejection fraction (%)	50 [30–60]	45 [35–52]	0.57
Modalities of ventilation			
Invasive mechanical ventilation (no, %)	11 (42 %)	6 (46 %)	0.91
Tidal volume (mL/kg)	6 [6–7]	7 [6–7]	0.34
PEEP (cmH_2_O)	7 [6–10]	7 [7–11]	0.22
Plateau pressure (cmH_2_O)	17 [16–19]	18 [16–24]	0.31
Respiratory rate (min^−1^)	20 [15–20]	20 [20–21]	0.71
Non-invasive mechanical ventilation (no, %)	6 (23 %)	0 (0 %)	0.16
No mechanical ventilation (no, %)	9 (35 %)	7 (54 %)	0.43
Acute respiratory distress syndrome (no, %)	10 (38 %)	6 (46 %)	0.89
Blood urea nitrogen (mmol/L)	32 [28–34]	30 [29–31]	0.25
Patients receiving norepinephrine (no, %)	9 (35 %)	7 (54 %)	0.43
Dose of norepinephrine (µg/kg/min)	0.15 [0.04–0.25]	0.24 [0.21–0.32]	0.69
Lactate (mmol/L)	1.1 [0.9–1.1]	0.9 [0.9–1.1]	0.38
Days in the ICU before inclusion (days)	13 [6–28]	11 [5–18]	0.67

Values are expressed as median [interquartile range] or number and frequency in  %

There was no significant difference between patients with and patients without intradialytic hypotension

*PEEP* positive end-expiratory pressure; *RRT* renal replacement therapy

**Table 2:** Settings of intermittent renal replacement therapy at baseline

	Patients without intradialytic hypotension	Patients with intradialytic hypotension	*p* value
	(*n* = 26)	(*n* = 13)	
Planned duration (h)	4.0 [4.0–5.3]	6.0 [4.0–6.0]	0.08
Rate of fluid removal (mL/h)	1000 [660–1000]	660 [500–1000]	0.53
Dialysate sodium concentration (mmol/L)	145 [144–145]	145 [144–145]	0.40
K^+^ in dialysate (mmol/L)	3.0 [2.0–3.0]	3.0 [3.0–3.0]	0.53
Temperature (°C)	36 [36–36]	36 [36–36]	–

Values are expressed as median [interquartile range] or number and frequency in %

There was no significant difference between patients with and patients without intradialytic hypotension

*RRT* renal replacement therapy

### Outcome of RRT

At mixed-effects model analysis, the global end-diastolic volume decreased in patients with and without intradialytic hypotension with similar slopes (−11 and −22 mL/m^2^/h, respectively, *p* = 0.40). Apart from global end-diastolic volume, in the 26 patients without intradialytic hypotension, no significant change in haemodynamic variables was observed during RRT (Table 3). In the 13 patients where intradialytic hypotension occurred, the time between the start of RRT and hypotension was 120 [60–180] min (Table 4). In these patients at H2, the decrease in CI (21 ± 15 %) was significantly lower than the decrease in arterial pulse pressure (11 ± 11 %) (*p* < 0.001). This was also the case for the decrease in stroke volume index (18 ± 16 %).

**Table 3:** Changes in haemodynamic variables

	Baseline	PLR (% change from baseline)		H1	H2	H3	H4	H5	H6
Number of patients at this time of the study									
Patients without intradialytic hypotension	26	26		26	26	25	23	8	7
Patients with intradialytic hypotension	13	13		13	9	5	2	0	0
Heart rate (beats/min)									
Patients without intradialytic hypotension	93 [81–106]	91 [79–101]	0 [-2–1]	90 [78–104]	93 [78–103]	91 [81–109]	89 [78–107]	97 [84–110]	97 [94–119.00]
Patients with intradialytic hypotension	95 [90–98]	95 [91–97]	0 [1–1]	91 [90–99]	93 [73–102]	89 [68–96]	103 [96–111]	–	–
Systolic arterial pressure (mmHg)*									
Patients without intradialytic hypotension	130 [120–151]	134 [120–151]	0 [0–3]	125 [115–153]	131 [118–148]	128 [113–150]	130 [110–149]	117 [109–140]	97 [94–119.00]
Patients with intradialytic hypotension	117 [102–131]	120 [111–130]	0 [0–3]	112 [108–120]	105 [102–108]	108 [98–109]	105 [92–119]	–	–
Mean arterial pressure (mmHg)									
Patients without intradialytic hypotension	87 [82–98]	90 [82–102]	0 [-1–3]	84 [78–95]	90 [80–96]	90 [76–96]	86 [79–94]	82 [74–92]	67 [66–61.00]
Patients with intradialytic hypotension	87 [79–89]	88 [82–92]	2 [-1–5]	81 [69–84]	75 [69–83]	68 [68–85]	78 [72–84]	–	–
Diastolic arterial pressure (mmHg)*									
Patients without intradialytic hypotension	64 [57–73]	64 [58–75]	0 [0–4]	62 [56–69]	65 [57–71]	65 [52–72]	61 [58–67]	63 [56–68]	57 [53–60.00]
Patients with intradialytic hypotension	65 [63–71]	68 [60–71]	1 [0–4]	63 [46–70]	59 [54–63]	51 [48–61]	62 [21–63]	–	–
Cardiac index (L/min/m^2^)*									
Patients without intradialytic hypotension	3.5 [2.7–4.2]	3.6 [2.9–4.1]	2 [0–5]	3.4 [2.6–3.8]	3.1 [2.6–3.8]	3.4 [2.2–3.8]	3.1 [2.6–3.7]	2.8 [2.5–3.4]	2.6 [2.4–2.9]
Patients with intradialytic hypotension	3.0 [2.3–3.2]	3.5 [3.1–4.0]	15 [11–36]	2.4 [1.7–2.8]	2.3 [1.6–2.3]	1.5 [1.4–2.1]	1.9 [1.5–2.2]	–	–
Global end-diastolic volume (mL/m^2^)									
Patients without intradialytic hypotension	765 [654–834]			716 [609–786]	685 [597–786]	707 [588–846]	712 [626–772]	812 [652–921]	865 [632–935]
Patients with intradialytic hypotension	700 [626–749]			693 [570–709]	641 [584–710]	500 [500–663]	539 [503–574]	–	–
Extravascular lung water (mL/kg)									
Patients without intradialytic hypotension	11 [8–16]			11 [8–15]	9 [8–15]	8 [8–14]	8 [7–12]	7 [6–9]	9 [6–11]
Patients with intradialytic hypotension	8 [7–14]			8 [7–15]	8 [7–13]	6 [5–7]	5 [5–6]	–	–
Cardiac function index (min^−1^)									
Patients without intradialytic hypotension	4.5 [3.5–5.4]			4.4 [4.1–5.2]	4.7 [3.9–5.6]	4.1 [3.8–5.1]	4.8 [4.0–5.0]	4.0 [3.8–4.1]	3.5 [3.0–4.4]
Patients with intradialytic hypotension	4 [3.5–5.0]			4.1 [2.9–5.0]	3.4 [2.5–4.1]	4.3 [3.4–5.2]	4.3 [4.0–4.5]	–	–
Volume of fluid removed (mL)									
Patients without intradialytic hypotension	–	–		1000 [660–1000]	2000 [1320–2000]	3000 [1860–3000]	4000 [2480–4000]	3300 [2900–5000]	4980 [3240–6000]
Patients with intradialytic hypotension	–	–		660 [500–1000]	2000 [1320–2000]	1980 [1500–3000]	3000 [2500–3500]	–	–
Body temperature									
Patients without intradialytic hypotension	37.6 [37.0–38.3]	–		37.6 [37.0–38.3]	37.6 [36.9–38.3]	37.3 [36.6–38.0]	37.2 [36.4–38.0]	37.2 [36.8–37.8]	37.2 [36.8–37.8]
Patients with intradialytic hypotension	37.5 [36.9–37.9]	–		37.5 [36.9–37.9]	37.4 [36.7–37.9]	37.6 [36.6–38.2]	37.0 [36.8–37.3]	–	–

Values were recorded before (baseline) and during PLR. Afterwards, they were recorded 1, 2, 3, 4, 5 and 6 h after starting renal replacement therapy (H1–H6)

Values are expressed as median [interquartile range] or number and frequency in  %

*PLR* passive leg raising

* *p* < 0 .05 for these variables, the slope from H1 to the end of renal replacement therapy or intolerance was significantly different from 1 and was higher in patients with no versus patients with intradialytic hypotension

**Table 4:** Haemodynamic variables at the time of intolerance to renal replacement therapy (*n* = 13)

	Before renal replacement therapy	At the previous record before intradialytic hypotension	At the time of intradialytic hypotension
Time elapsed since the onset of RRT (min)	–	120 [60–180]	130 [100–220]
Volume removed (mL)	–	1980 [1320–2000]	2000 [1000–3000]
Heart rate (beats/min)	95 [89–98]	91 [88–98]	97 [90–104]
Systolic arterial pressure (mmHg)	117 [102–131]	108 [100–111]	90 [76–95]
Mean arterial pressure (mmHg)	87 [79–89]	68 [66–76]	62 [52–65]*
Diastolic arterial pressure (mmHg)	65 [63–71]	57 [48–53]	47 [43–53]*
Cardiac index (L/min/m^2^)	3.0 [2.3–3.2]	2.1 [1.5–2.5]	1.7 [1.2–2.1]*
Global end-diastolic volume (mL/m^2^)	700 [626–749]	651 [500–728]	600 [554–619]
Extravascular lung water (mL/kg)	8 [7–14]	7 [6–8]	8 [7–12]
Cardiac function index (min^−1^)	4 [3.5–5.0]	3.7 [3.4–5.0]	3.4 [2.6–4.9]
Dose of norepinephrine (µg/kg/min) (in 5 patients)	0.24 [0.21–0.32]	0.24 [0.21–0.32]	0.24 [0.21–0.32]
Decision			
Interruption of RRT (no, %)	–	–	10 (77 %)
Increase of norepinephrine (no, %)	–	–	3 (23 %)

Values are expressed as median [interquartile range] or number and frequency in %

*RRT* renal replacement therapy

* *p* < 0.05 versus at the previous record before hypotension

The slopes of mean and diastolic arterial pressure and of CI differed significantly between patients with and without intradialytic hypotension. The slope differences in mean arterial pressure, diastolic arterial pressure, stroke volume index and CI were −4 mmHg/h, *p* = 0.02, −3 mmHg/h, *p* = 0.04, −2.2 L/min/m^2^, *p* < 0.01 and −0.17 L/min/m^2^/h, *p* = 0.02, respectively (Additional file 1: Table SDC2). No slope difference was found when comparing between-group changes in heart rate and systolic arterial pressure over time.

In 10 of the 13 patients with intradialytic hypotension, the decision was taken to stop RRT (Table 4). In these cases, blood restitution induced a significant increase in global end-diastolic volume, CI and mean arterial pressure (Additional file 1: Table SDC1). Norepinephrine was increased in three other patients with intradialytic hypotension. In all 13 cases, this treatment resolved hypotension.

### Differences at baseline between patients with and without intradialytic hypotension

**Fig. 1:**
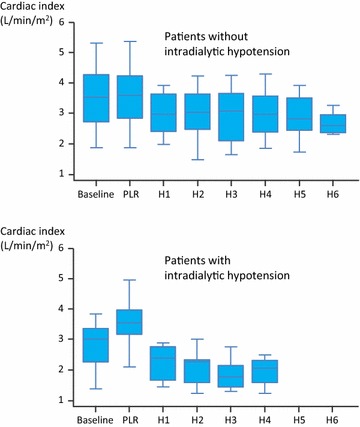
Changes in cardiac index at different study times in patients with and without intolerance to renal replacement therapy (RRT). *PLR* passive leg raising. The *central box* represents the values from lower to upper quartiles. The *middle line* represents the median. The *vertical line* extends from the minimum to the maximum values

The CI at baseline was significantly lower in patients with than in patients without intradialytic hypotension (3.0 [2.3–3.2] vs. 3.5 [2.7–4.2] L/min/m^2^, respectively, *p* = 0.03, Table 3; Fig. 1).

Patients with and without intradialytic hypotension did not differ at baseline in any variables except CI, including heart rate, systolic and mean arterial pressures, global end-diastolic volume, proportion of patients receiving norepinephrine and dose of norepinephrine in patients receiving this drug (Tables 1, 3). The settings of intermittent RRT at baseline were similar in both groups of patients (Table 2).

### Prediction of intradialytic hypotension

**Fig. 2:**
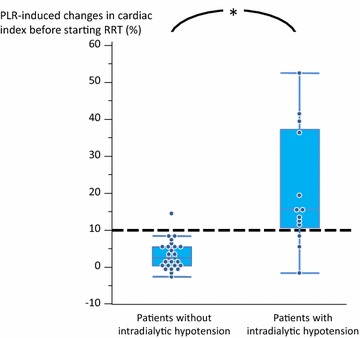
Changes in cardiac index induced by passive leg raising (PLR) test performed before starting renal replacement therapy (RRT) in patients with and without intradialytic hypotension. *p < 0.05 versus patients with intradialytic hypotension

As shown in Table 3 and Fig. 2, in the 26 patients without intradialytic hypotension, the PLR test did not induce any significant change in CI. Conversely, in patients with intradialytic hypotension, PLR significantly increased CI by 20 ± 16 % (*p* = 0.001, Table 3; Fig. 2).

**Fig. 3:**
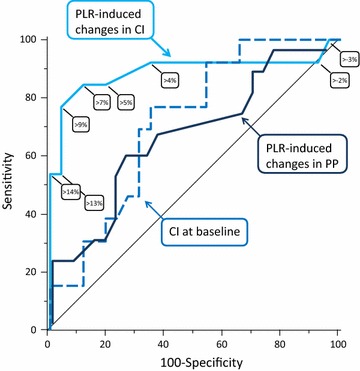
Receiver operating characteristic curves describing the ability of the changes in cardiac index (CI) induced by passive leg raising (PLR, with corresponding thresholds), the changes in arterial pulse pressure (PP) induced by PLR and the value of cardiac index at baseline to predict the occurrence of intradialytic hypotension

PLR-induced changes in CI predicted the occurrence of intradialytic hypotension with an AUC under the ROC curve of 0.89 (95 % confidence interval 0.75–0.97) (*p* < 0.05 from 0.50) (Fig. 3; Additional file 1: Table SDC3). The best diagnostic threshold was 9 %. It provided a sensitivity of 77 % (95 % confidence interval 46–95 %), a specificity of 96 % (95 % confidence interval 80–100 %), a positive predictive value of 91 % (95 % confidence interval 57–100 %) and a negative predictive value of 89 % (95 % confidence interval 72–98 %). Three false negatives were observed. In these patients, the volume of fluid that had been removed was 2420, 2750 and 3500 mL.

As shown in Fig. 3 and Additional file 1: Table SDC3, the area under the ROC curves constructed for the value of CI, stroke volume and global end diastolic at baseline was significantly lower than that of PLR-induced changes in CI. This was also the case for the PLR-induced changes in arterial pulse pressure (Fig. 3; Additional file 1: Table SDC3).

## Discussion

In this study, we observed that preload dependence, as assessed by the PLR test, was higher in patients who presented haemodynamic intolerance to fluid removal during intermittent RRT than in patients who well tolerated fluid removal. A PLR-induced increase in CI at baseline by more than 9 % predicted intradialytic hypotension with a good specificity and a good positive predictive value.

Excessive fluid removal is the main cause of intradialytic hypotension [9, 25]. Based on physiology, one could make the hypothesis that this impairment may occur in the specific condition of preload responsiveness, i.e. if the heart is working on the steep and initial part of the cardiac function curve [26]. In such conditions, a decrease in preload induces a decrease in cardiac output and then in blood pressure.

Consistent with this hypothesis, we observed that the risk of intradialytic hypotension during fluid removal was associated with the presence of preload dependence at baseline, while the rate of fluid removal was similar in patients with and without intradialytic hypotension. Moreover, a positive PLR test before RRT identified patients who were likely to present intradialytic hypotension. A 9 % increase in CI during the PLR test was the threshold providing the best Youden index. Clinicians may choose the threshold depending on whether they want to favour sensitivity or specificity (Fig. 3).

Nowadays, in order to decide to undertake ultrafiltration or not, clinicians likely take into account some criteria like baseline levels of arterial pressure, cardiac output, heart rate, cardiac preload (assessed by the global end-diastolic volume in our study). They also likely take into account the fact that the patient receives norepinephrine and the dose of norepinephrine. What our study shows is that intradialytic hypotension was not predicted by any of these criteria that are commonly used. Except the PLR-induced changes in CI, CI was the only variable that differed at baseline between patients with and without intradialytic hypotension. Nonetheless, we found that the prediction of intolerance to fluid removal that it provided was significantly poorer than that provided by the PLR test. It was the same for stroke volume index. The global end-diastolic volume did neither predict intradialytic hypotension. This is consistent with the fact that, although it is a reliable marker of preload [15, 27–29], it does not detect preload responsiveness [24, 28, 30]. These facts suggest that our study actually brings a valuable answer to a common question remained pending until now.

Our results suggest that the PLR test can be used to guide fluid removal in critically ill patients. The positive predictive value and the specificity were good, meaning that a positive PLR test incites to not remove fluid. For a 9 % increase in CI, the PLR test predicted intradialytic hypotension with a good specificity but a lower sensitivity, especially with false-negative cases. An explanation could be that the ability of the PLR test to detect preload responsiveness is not perfect. Another explanation could be that preload responsiveness may be absent at baseline in some patients but could appear later, along with fluid removal. Accordingly, the volume of fluid removed was particularly high in the three false positives we observed. A third explanation for the false negatives could be that, besides excessive fluid removal, vasodilation may contribute to some cases of intradialytic hypotension [9, 25].

Our study should be regarded as a proof of the concept that the risk of intradialytic hypotension in patients with acute circulatory failure is associated with the presence of preload dependence. In a next step, prospective studies should compare strategies where the decision to remove fluid during RRT would be based on the assessment of preload dependence or not, with the occurrence of intradialytic hypotension as an end point. More generally, this would offer the opportunity to decide fluid removal on standardised protocols. In this regard, an interesting study showed that protocolised fluid removal with continuous RRT was associated with large negative fluid balances in patients, while standard fluid balance prescription did not result in a substantial negative fluid balance [31].

We chose to investigate fluid removal performed only by intermittent RRT and not by other means. Indeed, with diuretics, the volume of removed fluid is variable and this would make the investigation complex. Also, we did not test continuous RRT since it is not in our practice to use this technique in haemodynamically stable patients. The majority of our patients were in the resolving phase of circulatory failure. Even though our findings should be the same at earlier phases of shock, the decision to reduce cardiac preload is less often taken at the initial phase of haemodynamic resuscitation.

We defined intolerance to fluid removal on changes in mean arterial pressure. Nevertheless, since the sympathetic system tends to maintain mean arterial pressure stable when CI changes [32], changes in CI occurred earlier than changes in mean arterial pressure in our patients. For instance, at H2 in patients with intradialytic hypotension, the decrease in mean arterial pressure was 14 ± 11 %, while the decrease in CI was 21 ± 15 % (*p* < 0.05). Assessing whether haemodynamic intolerance to fluid removal should be defined on CI rather than on mean arterial pressure was out of the purpose of this study.

We decided to leave the ultrafiltration rate at the discretion of the attending clinician. In theory, one could suspect that patient characteristics at baseline would have influenced this choice. Nevertheless, the study protocol stipulated that the choice of ultrafiltration rate should be taken before the PLR test and should not be changed afterwards. Also, it is unlikely that other factors like heart rate, the presence of norepinephrine infusion and the rate of this infusion for instance would have significantly influenced the choice of ultrafiltration rate. Indeed, in such a case, such characteristics would have been significantly different between patients with and without intradialytic hypotension. Except for CI at baseline, this was not the case.

One limitation of the present research is that, for feasibility reasons, we did not perform the PLR test at each time point of the study. It is likely that a continuous assessment of preload dependence would avoid the occurrence of intradialytic hypotension, but this has yet to be investigated. Another limitation is that we did not monitor the changes in blood urea nitrogen or plasma sodium concentration during dialysis, while their variations may influence the haemodynamic tolerance of dialysis [9]. Nevertheless, the facts that their levels at baseline were similar in both groups and that the rate of dialysate flow was also similar suggest that the changes of these variables were similar in patients with and without intradialytic hypotension. A limitation of our study could also reside in the fact that we did not measure the intra-abdominal pressure, although it may have an impact on the phenomena we observed [33, 34]. Another limitation is that the PLR test was not performed in a blind manner. Finally, in this study, we did not compare two strategies of fluid removal, one in which it would be guided by the PLR test and another one in which it would not. This should be performed by further investigations.

In conclusion, the presence of preload dependence, as assessed by a positive PLR test before starting RRT, predicts whether fluid removal by ultrafiltration will induce haemodynamic intolerance or not.

## Key messages

- Haemodynamic intolerance to fluid removal during RRT is associated with the presence of preload dependence.
- A positive PLR test before fluid removal predicts the occurrence of subsequent hypotension during RRT.

## Additional file

10.1186/s13613-016-0149-1 Tables SDC1, SDC2 and SDC3.

## Abbreviations

CI: cardiac index
PLR: passive leg raising test
RRT: renal replacement therapy
