# Supplementary material for: The passive leg raising test to guide fluid removal in critically ill patients
Source: Ann Intensive Care. 2016 May 20;6:46. doi: 10.1186/s13613-016-0149-1 (PMC4875574; doi:10.1186/s13613-016-0149-1)
Supplement: Supplementary file 1 — 10.1186/s13613-016-0149-1 Tables SDC1, SDC2 and SDC3. [file 13613_2016_149_MOESM1_ESM.docx]

# Supplemental Digital Content

# The passive leg raising test to guide fluid removal in critically ill patients

***Authors***

Xavier MONNET, MD, PhD^1, 2^

xavier.monnet@aphp.fr

Flora CIPRIANI, MD^1, 2^

flora.cipriani@yahoo.com

Laurent CAMOUS, MD^1, 2^

laurent_lau@hotmail.com

Pierre SENTENAC, MD^1, 2^

pierre.sentenac@orange.fr

Martin DRES, MD^1, 2^

martin.dres@aphp.fr

Evguenia KRASTINOVA, MD, PhD^2, 3^

krastinova@gmail.com

Nadia ANGUEL, MD^1, 2^

nadia.anguel@aphp.fr

Christian RICHARD, MD^1, 2^

christian.richard@aphp.fr

Jean-Louis TEBOUL, MD, PhD^1, 2^

jean-louis.teboul@aphp.fr

***Addresses***

1. Hôpitaux universitaires Paris-Sud, Hôpital de Bicêtre, service de réanimation médicale, 78, rue du Général Leclerc, Le Kremlin-Bicêtre, F-94270 France
2. Univ Paris-Sud, Faculté de médecine Paris-Sud, Inserm UMR S_999, Le Kremlin-Bicêtre, 63, rue Gabriel Péri, F-94270 France
3. Hôpitaux universitaires Paris-Sud, Hôpital de Bicêtre, Service de santé publique, 78, rue du Général Leclerc, Le Kremlin-Bicêtre, F-94270 France

***Corresponding author***

Prof. Xavier Monnet

Service de réanimation médicale

Centre Hospitalier Universitaire de Bicêtre

78, rue du Général Leclerc

94 270 Le Kremlin-Bicêtre

France

e-mail: xavier.monnet[@bct.aphp.fr](mailto:jean-louis.teboul@bct.ap-hop-paris.fr)

Phone: + 33 1 45 21 35 39

Fax: + 33 1 45 21 35 51

**Table SDC2. Changes in haemodynamic variables according to tolerance vs. intolerance to renal replacement therapy, using the linear mixed-effects models.**

| **Variable** | **Estimate** | **SE** | ***p* value*** |
| --- | --- | --- | --- |
| **Intercept for MAP in cases without intolerance to RRT** | 88.45 | 2.57 | <0.0001 |
| Slope value of MAP in cases without intolerance to RRT (mmHg/h) | -0.33 | 0.76 |  |
| Slope value of MAP in cases with intolerance to RRT (mmHg/h) | -4.54 | 1.59* |  |
| **Intercept for CI in cases** **without intolerance to RRT** | 3.30 | 0.19 | <0.001 |
| Slope value of CI in cases without intolerance to RRT (L/min/m^2^/h) | -0.09 | 0.03 |  |
| Slope value of CI in cases with intolerance to RRT(L/min/m^2^/h) | -0.27 | 0.07* |  |
| **Intercept for stroke index in cases without intolerance to RRT** | 38.90 | 2.58 | <0.001 |
| Slope value of stroke index in cases without intolerance to RRT (mL/m^2^/h) | -0.86 | 0.37 |  |
| Slope value of stroke index in cases with intolerance to RRT(mL/m^2^/h) | -3.04 | 0.76* |  |
| **Intercept for DAP in cases without intolerance to RRT** | 65.65 | 2.64 | <0.001 |
| Slope value of DAP in cases without intolerance to RRT (mmHg/h) | -0.70 | 0.58 |  |
| Slope value of DAP in cases with intolerance to RRT (mmHg/h) | -3.39 | 1.18* |  |

DAP: diastolic arterial pressure, MAP: mean arterial pressure, CI: cardiac index, RRT: renal replacement therapy.

***** p<0.05 Slope value in cases without intolerance to RRT vs. in cases with intolerance to RRT.
